# Proof of concept for monoclonal antibody therapy in a cellular model of acquired long QT syndrome type 3

**DOI:** 10.1152/ajpheart.00628.2023

**Published:** 2023-11-10

**Authors:** Lenke Kis, Jin Li

**Affiliations:** ^1^Center for Translational and Experimental Cardiology, Department of Cardiology, University of Zurich, Zurich, Switzerland; ^2^Department of Cardiology, University Heart Center, University Hospital Zurich, Zurich, Switzerland

**Keywords:** anemone toxin, antibody therapy, cardiac arrhythmia, human induced pluripotent stem cell-derived cardiomyocyte, long QT syndrome

## Abstract

Long QT syndrome (LQTS) type 3 although less common than the first two forms, differs in that arrhythmic events are less likely triggered by adrenergic stimuli and are more often lethal. Effective pharmacological treatment is challenged by interindividual differences, mutation dependence, and adverse effects, translating into an increased use of invasive measures (implantable cardioverter-defibrillator, sympathetic denervation) in patients with LQTS type 3. Previous studies have demonstrated the therapeutic potential of polyclonal KCNQ1 antibody for LQTS type 2. Here, we sought to identify a monoclonal KCNQ1 antibody that preserves the electrophysiological properties of the polyclonal form. Using hybridoma technology, murine monoclonal antibodies were generated, and patch clamp studies were performed for functional characterization. We identified a monoclonal KCNQ1 antibody able to normalize cardiac action potential duration and to suppress arrhythmias in a pharmacological model of LQTS type 3 using human-induced pluripotent stem cell-derived cardiomyocytes.

**NEW & NOTEWORTHY** Long QT syndrome is a leading cause of sudden cardiac death in the young. Recent research has highlighted KCNQ1 antibody therapy as a new treatment modality for long QT syndrome type 2. Here, we developed a monoclonal KCNQ1 antibody that similarly restores cardiac repolarization. Moreover, the identified monoclonal KCNQ1 antibody suppresses arrhythmias in a cellular model of long QT syndrome type 3, holding promise as a first-in-class antiarrhythmic immunotherapy.

## INTRODUCTION

Congenital long QT syndrome (LQTS) is one of the leading causes of sudden cardiac death below the age of 20 and has an estimated prevalence of 1:2,000 (1). Despite extensive research on LQTS, there is a mismatch between the rapidly evolving understanding of the genetic architecture and the slow progress in the care and treatment of patients with LQTS (2). Present-day choices for pharmacological therapy can be suboptimal in regard to providing adequate efficacy and safety for a subset of patients with LQTS (3, 4). This is especially true for patients with LQTS type 3 with a mutation in the *SCN5A* gene leading to increased persistent *I*_Na_ across the Na_v_1.5 sodium channel, who remain at higher risk throughout life (3, 5, 6). It seems that antiarrhythmic drugs (flecainide, mexiletine, ranolazine) were often overlooked in favor of interventional (left sympathetic cardiac denervation) and device therapy (implantable cardioverter-defibrillator, ICD; 7–9). Although these approaches save lives, they are invasive in nature and carry a risk for procedural complications that can impact quality of life (10–12). In the search to overcome this unmet need for a more targeted and effective pharmacological treatment of LQTS, we previously identified polyclonal antibodies targeting the cardiac KCNQ1 potassium channel, with the potential to correct arrhythmogenicity in a cellular model of LQTS type 2 (3, 13). The novelty brought by the study was the mechanism of action aiming at modulating an unaffected ion channel (increased K^+^ outflow through KCNQ1 channels, *I*_Ks_ the slow component of the delayed rectifier K^+^ current) to compensate for the loss-of-function of the mutated one (decreased K^+^ outflow through hERG channels, *I*_Kr_ the rapid component of the delayed rectifier K^+^ current; 3). In the effort to refine this novel concept of antiarrhythmic immunotherapy, a murine monoclonal antibody was generated and tested for its therapeutic potential for LQTS using human-induced pluripotent stem cell-derived (iPSC) cardiomyocytes.

## MATERIALS AND METHODS

### Monoclonal Antibody Production

Balb/c mice were immunized against the KCNQ1 channel peptide sequence using standard protocol (ProteoGenix, France; 3, 13, 14). Briefly, mice received weekly subcutaneous injections of KCNQ1 peptide supplemented with Freund’s adjuvant. After four weeks, spleen cells from mice with the highest antibody titer were collected and fused with myeloma cells (ProteoGenix, France). Hybridoma cells were cultured in RPMI 1640-1% l-glutamine supplemented with 10% fetal bovine serum and 1% penicillin-streptomycin. Sequential separation of cells of different passages was performed using hypoxanthine-aminopterin-thymidine (HAT) and medium. Through repetitive subcloning by the limiting dilution technique and screening via enzyme-linked immunosorbent assay (ELISA), six clones were identified that specifically produce IgGs targeting the KCNQ1 channel sequence. Six monoclonal antibodies were produced from the selected clones and purified on protein G columns (ProteoGenix, France). Monoclonal antibodies were collected in PBS, and concentration was determined with the A280 method.

### CHO Cell Culture and Patch Clamp Recording

Chinese hamster ovary (CHO) cells stably expressing human KCNQ1/KCNE1 channels were used to select the candidate monoclonal antibody for downstream application (3). Briefly, cells were cultured in Ham’s F-12 nutrient mix supplemented with 10% fetal bovine serum and 1% penicillin/streptomycin. For patch clamp experiments, cells were plated onto Petri dishes (1,000–2,000 cells/cm^2^) in culture medium ± monoclonal antibody (30 µg/mL). An EPC-10 amplifier controlled by PATCHMASTER (HEKA Elektronik, Germany) was used to record *I*_Ks_ in the whole cell configuration at room temperature. The following external solution was used (in mmol/L): 140 NaCl, 5 KCl, 1 MgCl_2_, 10 HEPES, 1.8 CaCl_2_, and 10 glucose ± monoclonal antibody. Borosilicate glass capillaries (Harvard Apparatus, tip resistances 5–7 MΩ) were filled with internal solution composed of (in mmol/L): 100 K^+^ aspartate, 20 KCl, 2 MgCl_2_, 1 CaCl_2_, 10 EGTA, 5 K^+^ ATP, 10 HEPES, and 40 glucose. *I*_Ks_ were measured by holding the cells at −60 mV and applying depolarizing test pulses (3,000 ms, 0.1 Hz) from −50 mV to +70 mV in 10-mV incremental steps, followed by repolarizations (2,000 ms) to −40 mV. *I*_Ks_ were low-pass filtered at 2.9 kHz and sampled at 4 kHz. Data were analyzed with FITMASTER (HEKA Elektronik, Germany). Activation and inactivation curves were fit with a Boltzmann function: *I*/*I*_max_ = 1/(1 + e^(*V*^^1/2−^^*Vt*)/^^*k*^), where *V*_1/2_ is half-maximal activation potential, *V*_t_ is test pulse potential, and *k* is slope factor (3).

### Antibody Kinetics and Affinity Measurement

A Biacore 8 K surface plasmon resonance (SPR) instrument (GE Healthcare Life Sciences, ProteoGenix, France) equipped with a CM5 sensor chip was used to generate binding kinetic rate and affinity constants. The monoclonal antibody (10 µg/mL) was immobilized onto the CM5 chip by amide coupling, following manufacturer’s protocol. Different concentrations of monoclonal antibody (7.8125–500 nM, twofold serial dilutions) were injected at a flow rate of 30 µL/min for 120 s, followed by a dissociation phase of 600 s. Injections were performed in triplicate to assess for the assay’s reproducibility. Data were analyzed with Biacore 8 K Evaluation Software.

### High-Resolution Conformational Epitope Mapping

Mapping of antibody epitopes was performed on PEPperCHIP by PEPperPRINT GmbH, Germany, covering the full-length sequence of KCNQ1 protein (NP_000209.2) elongated with neutral GSGSGSG linkers at the COOH- and NH_2_-terminus to avoid truncated peptides. The elongated antigen sequence was translated into 7, 10, and 13 overlapping amino acid peptides, cyclized via a thioether linkage between a C-terminal cysteine and an appropriately modified NH_2_-terminus. The resulting conformational KCNQ1 peptide microarrays contained 2,043 different peptides printed in duplicate and framed by additional hemagglutinin (YPYDVPDYAG, 134 spots) control peptides. The monoclonal antibody was incubated at 0.1 µg/mL. Goat anti-mouse IgG (H + L) DyLight680 served as a secondary antibody. LI-COR Odyssey Imaging System was used for scanning. Quantification of spot intensities and peptide annotation were performed with PepSlide analyzer. Based on averaged median foreground intensities, an intensity map was generated. A maximum spot-to-spot deviation of 40% was tolerated, otherwise, the corresponding intensity value was zeroed.

### Human iPSC-Cardiomyocyte Culture and Patch Clamp Recording

Human iPSC-derived ventricular cardiomyocytes (Pluricyte, Ncardia, The Netherlands) were cultured according to manufacturer’s instructions (3). Human iPSC-cardiomyocytes were plated at a density of 25,000 cells/cm^2^ on Petri dishes coated with Corning Matrigel. Spontaneous action potentials were recorded between *days 7* and *14* postthawing, under current-clamp conditions with EPC-10 amplifier (PATCHMASTER) at 37°C, as previously described (3). The external solution was composed of (in mmol/L): 140 NaCl, 5 KCl, 1 MgCl_2_, 10 HEPES, 1.8 CaCl_2_, and 10 glucose ± monoclonal antibody. To induce LQTS type 2 in human iPSC cardiomyocytes pharmacologically, cells were exposed to the selective hERG blocker, 10 nM E-4031 (Alomone Labs, Israel), and recordings began after 30 min of incubation. To simulate the electrical phenotype of LQTS type 3, late *I*_Na_ was selectively increased using sea anemone toxin 2 (5 nM ATX-II, Alomone, Israel) after 5 min of incubation. Borosilicate glass pipettes had tip resistances of 2–4 MΩ. The internal solution contained (in mmol/L) 110 K^+^ aspartate, 20 KCL, 1 MgCl_2_, 5 Mg^2+ ^ATP, 0.1 Li^+ ^GTP, 10 HEPES, 5 Na^+^ phosphocreatine, 0.05 EGTA, and 200 µg/mL amphotericin. Data were analyzed with FITMASTER. The action potential duration (APD) was determined at 90% (APD_90_) repolarization.

### Statistics

Results are shown as means ± SE. All data underwent Shapiro–Wilk test to assess for the normality of distribution. Statistical differences between groups with normally distributed data were determined by one-way analysis of variance (ANOVA) followed by Tukey’s multiple comparison post hoc test. For comparisons between two group means, two-tailed Student’s *t* test was applied to determine the statistical significance of normally distributed data. In the case of nonnormal distribution, Mann–Whitney *U* test was used. GraphPad Prism 7 software (GraphPad software) was used for statistical analyses. A *P* value of <0.05 was considered statistically significant.

## RESULTS

### Selection of Functional Monoclonal KCNQ1 Antibody

**Figure 1.**
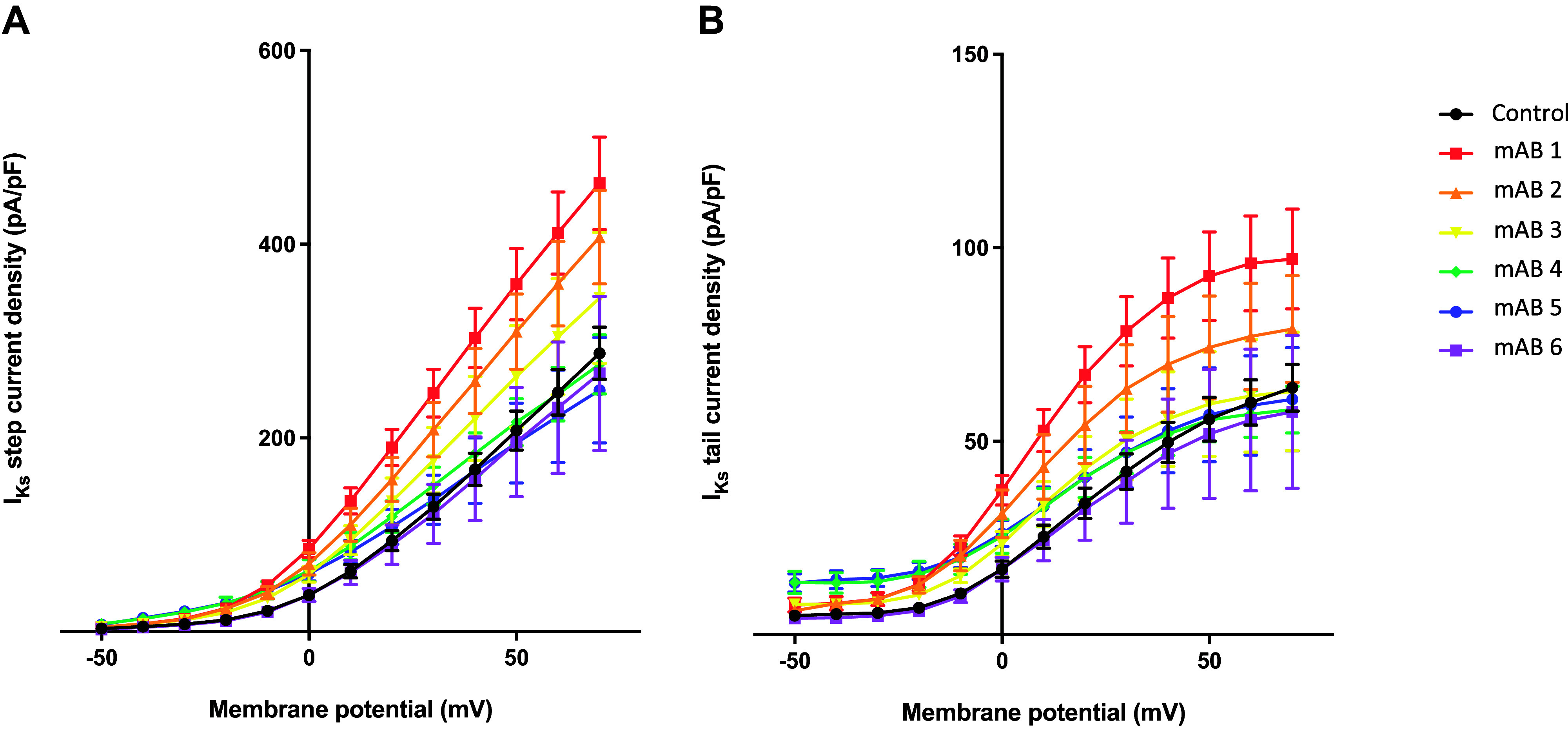
Effect of six different monoclonal KCNQ1 antibodies on slow component of the delayed rectifier K^+^ current (*I*_Ks_). Represented are *I*_Ks_ step (*A*) and tail (*B*) current densities as a function of the test potential comparing control cells (*n* = 16, mean cell capacitance 17.2 ± 2.2 pF) and cells treated with 30 µg/mL monoclonal antibodies: mAB-1 (*n* = 15, 16.7 ± 1.1 pF), mAB-2 (*n* = 12, 18.2 ± 1.9 pF), mAB-3 (*n* = 8, 18.4 ± 2.5 pF), mAB-4 (*n* = 8, 20.8 ± 3.4 pF), mAB-5 (*n* = 8, 17.7 ± 1.9 pF), and mAB-6 (*n* = 5, 26.0 ± 6.3 pF).

**Figure 2.**
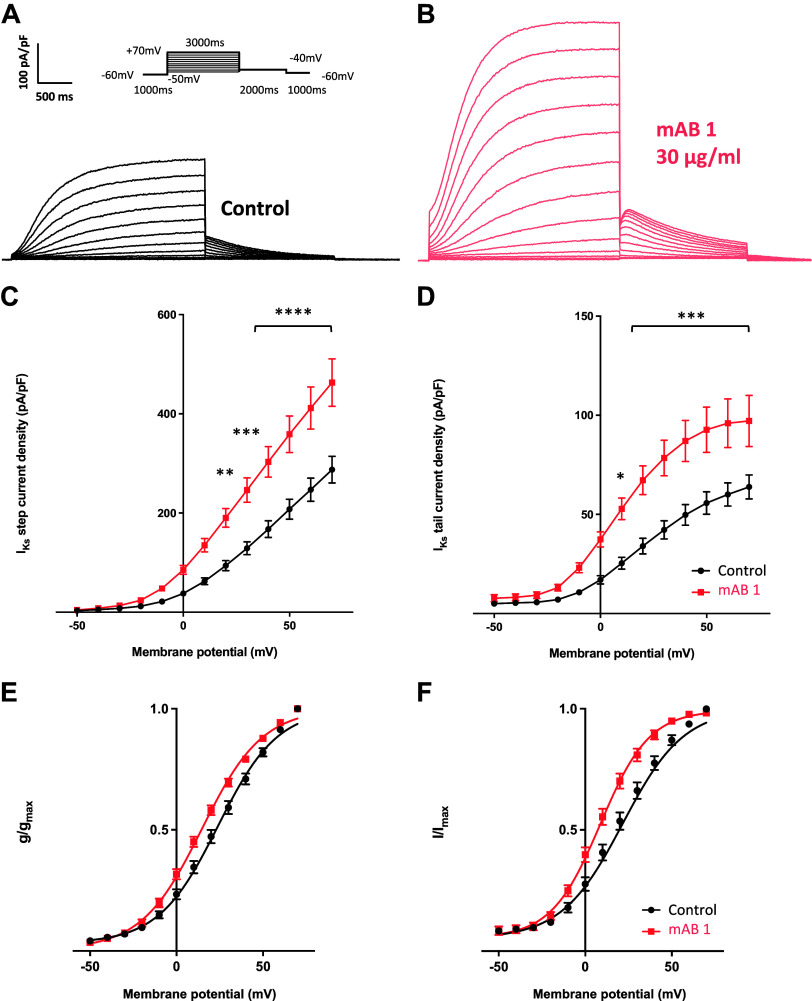
Effect of mAB-1 monoclonal antibody on slow component of the delayed rectifier K^+^ current (*I*_Ks_). Representative *I*_Ks_ traces recorded in Chinese hamster ovary (CHO) cells under control condition (*A*; cell capacitance, 13.7 pF) and in the presence of 30 µg/mL mAB-1 (*B*; 13.6 pF). *C* and *D*: *I*_Ks_ step and tail current densities as a function of the test potential comparing control cells (*n* = 16) and cells treated with mAB-1 (*n* = 15). *P < 0.05, **P < 0.01, ***P < 0.001, ****P < 0.0001. *E*: voltage-dependent activation of *I*_Ks_. In the presence of mAB-1, *I*_Ks_ were activated at more negative potentials compared with control cells (*P* < 0.0001), without manifest effect on the slope factor *k* [control, half-maximal activation potential (*V*_1/2_) = 23.6 ± 1.3 mV, *k* = 16.9 ± 1.2 mV; mAB-1, *V*_1/2 _= 14.6 ± 0.9 mV, *k* = 17.5 ± 1.0 mV]. *F*: voltage-dependent deactivation of *I*_Ks_. mAB-1 led to a leftward shift of the voltage-dependence of deactivation (*P* < 0.0001), whereas not affecting the slope factor *k* (control, *V*_1/2 _= 20.0 ± 1.4 mV, *k* = 15.9 ± 1.4 mV; mAB-1, *V*_1/2 _=_ _8.7 ± 1.1 mV, *k* = 14.2 ± 1.1 mV).

Hybridoma supernatants were tested by ELISA to ensure that the secreted antibody retained specificity for the KCNQ1 channel peptide. Out of 40 hybridoma clones, six producing functional IgG antibodies with specificity were identified (mAB 1–6). We performed patch-clamp experiments to study the effects of all six monoclonal antibodies on *I*_Ks_ in CHO cells stably expressing human *I*_Ks_ channels. As illustrated in Fig. 1, mAB-1 best replicated the effect of the polyclonal antibody population: mAB-1 increased the mean *I*_Ks_ step current by 1.6-fold at +70 mV, and the mean *I*_Ks_ tail current by 1.5-fold upon repolarization to −40 mV (Fig. 2, *A*–*D*). Analogous to the polyclonal KCNQ1 antibodies, mAB-1 shifted the half-maximal activation potential (*V*_1/2_) by −9 mV, whereas shifting the voltage-dependence of deactivation to more negative potentials by 11 mV (Fig. 2, *E* and *F*). The activation and deactivation slope factors *k* reflecting voltage sensitivity remained unchanged.

### Characterization of Candidate Monoclonal KCNQ1 Antibody

**Figure 3.**
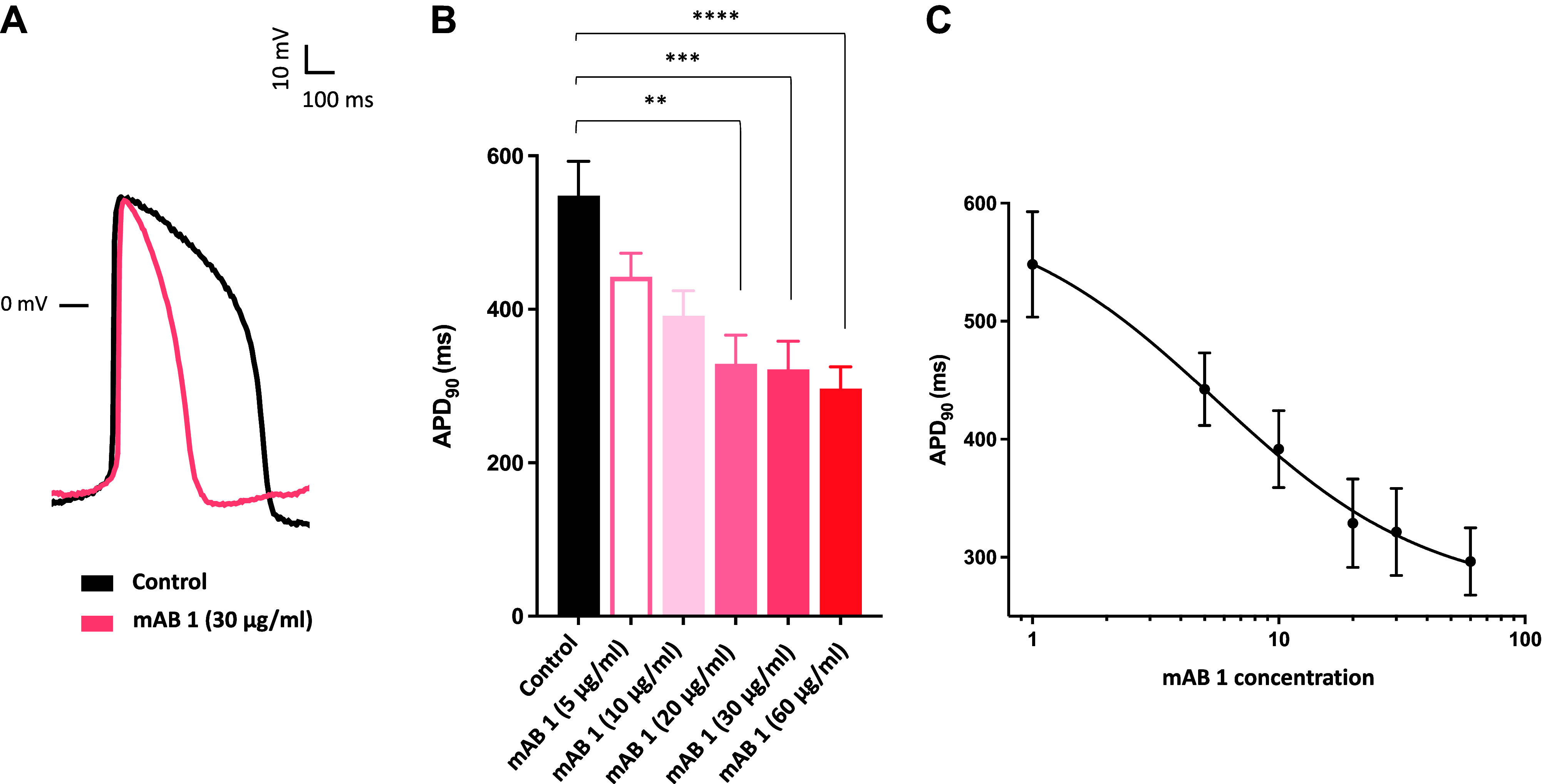
Effect of mAB-1 monoclonal antibody on human iPSC-cardiomyocytes (hiPSC-CMCs). *A*: representative action potential traces recorded in hiPSC-CMCs under control condition and in the presence of 30 µg/mL mAB-1. *B*: bars represent mean action potential duration at 90% repolarization (APD_90_) of control cells (*n* = 15) and cardiomyocytes treated with 5 µg/mL (*n* = 4), 10 µg/mL (*n* = 10), 20 µg/mL (*n* = 11), 30 µg/mL (*n* = 14), and 60 µg/mL (*n* = 14) mAB-1. **P < 0.01, ***P < 0.001, ****P < 0.0001. *C*: concentration-response curve for the absolute APD_90_ reduction effect at five different concentrations of mAB-1. A sigmoidal curve fit to the data determined an EC_50_ of 5.7 µg/mL (*R*^2^ = 0.9967).

We next tested the effect of mAB-1 on cardiac repolarization at various concentrations (Fig. 3, *A* and *B*). Action potential parameters derived from human iPSC-cardiomyocytes are delineated in Supplemental Table S1. We observed a concentration-dependent shortening of APD_90_ by the monoclonal antibody with a sigmoidal concentration-response relationship (Fig. 3*C*). The half-maximal effective concentration (EC_50_) was calculated at 5.7 µg/mL mAB-1. The kinetics of monoclonal antibody binding to the respective KCNQ1 peptide was determined with SPR technique (Supplemental Fig. S1). A mean equilibrium dissociation constant (*K*_D_) of 9.02 × 10^−9^ ± 6.99 × 10^−10^ M was calculated. The identified monoclonal antibody mAB-1 thus exhibits an overall high affinity with *K*_D_ values in the nanomolar range. Conformational epitope mapping consistently revealed a very strong antibody response against epitope-like spot patterns formed by adjacent peptides with the consensus motif VEFG (a sequence corresponding to our targeted epitope, the extracellular loop between the fifth and sixth transmembrane domain of KCNQ1; Supplemental Fig. S2).

### Therapeutic Potential of Candidate Monoclonal KCNQ1 Antibody In Vitro

**Figure 4.**
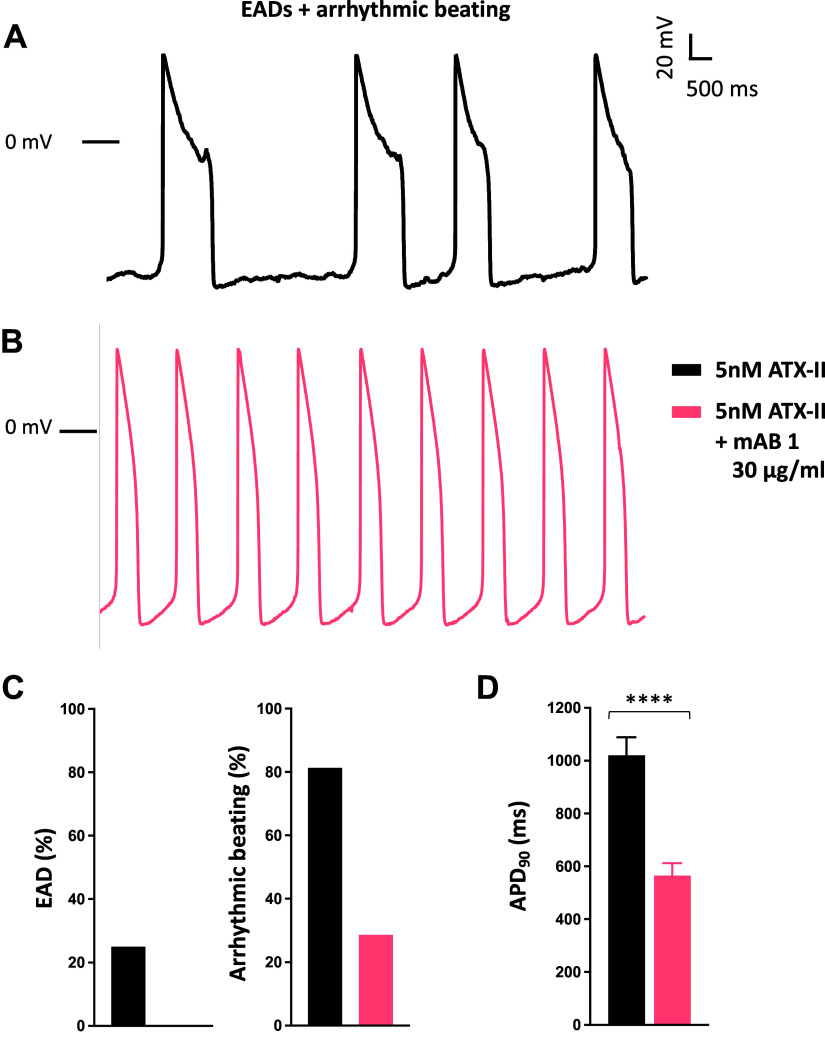
Effect of mAB-1 monoclonal antibody on human iPSC-cardiomyocytes (hiPSC-CMCs) in the context of pharmacological LQTS type 3. *A*: representative action potentials recorded in hiPSC-CMCs challenged with sea anemone toxin 2 (ATX-II) leading to early afterdepolarizations (EADs) and arrhythmic beating. *B*: representative action potentials recorded in hiPSC-CMCs treated with 30 µg/mL mAB-1 and challenged with ATX-II. Bars represent the incidence of EADs and arrhythmic beating (*C*) and mean APD_90_ (*D*) of hiPSC-CMCs challenged with ATX-II (*n* = 16) ± 30 µg/mL mAB-1 (*n* = 14). ****P < 0.0001.

To confirm that the previously observed therapeutic effect of polyclonal KCNQ1 antibodies is reproducible with the identified monoclonal candidate, we tested mAB-1 on human iPSC-cardiomyocytes with a drug-induced LQTS type 2 phenotype. As expected, the selective *I*_Kr_ inhibitor, E-4031, caused a fourfold increase in APD_90_ (Supplemental Table S2) and gave rise to frequent early afterdepolarizations (EADs, 71.4%, Supplemental Fig. S3). Incubation of cells with mAB-1 significantly shortened APD_90_ and completely blunted arrhythmic events, thus validating its efficacy (Supplemental Fig. S3 and Supplemental Table S2). We then explored the therapeutic potential of monoclonal antibody for LQTS type 3. To that end, we studied the effect of mAB-1 in a pharmacological model of human iPSC-cardiomyocytes using ATX-II that selectively disrupts the inactivation mechanism of Na_v_1.5 in a way that mimics LQTS type 3 (15–17). When ATX-II was applied, APD_90_ in our cells prolonged by twofold (mean APD_90_ from 548.2 ± 44.7 to 1,021.0 ± 67.2 ms, *P* < 0.0001). Consequently, ATX-II triggered EADs (25%) as well as arrhythmic beating (81.3%, Fig. 4*A*). In contrast, cells pretreated with mAB-1 exhibited APD_90_ normalized to baseline values (mean APD_90_, 564.9 ± 47.3 ms, *P* < 0.0001, Fig. 4, *B*–*D*, Supplemental Table S3). Moreover, mAB-1 treatment completely suppressed EADs and reduced arrhythmic beating (Fig. 4*C*).

## DISCUSSION

Since the first description and empirical treatment of LQTS with digoxin, the main advancement that has been implemented to prevent arrhythmias was the use of beta-blockers, whereas ICDs and left cardiac sympathetic denervation were mainly applied in cases of recurrent arrhythmias (18). Despite their effectiveness, current standard of care has limitations, and a subgroup of patients with LQTS, in particular subtype 3 LQTS, remain incompletely protected by antiadrenergic measures (19). This unmet medical need continues to motivate the search for better pharmacological treatment. First attempts on patients with LQTS type 3 were limited to small case series using drugs already on the market but approved for other indications. Examples include ranolazine as an antianginal agent, flecainide, and mexiletine as antiarrhythmic drugs (7–9). They all have in common to inhibit late Na^+^ current. However, the differential sensitivity (mutation-dependent efficacy), nonspecific nature (concomitant block of peak Na^+^ current by flecainide), and off-target effects (e.g., *I*_Kr_ inhibition by ranolazine and flecainide) are major concerns narrowing their therapeutic window (20). Eleclazine is the latest generation Na^+^ channel blocker. Although it went to phase III clinical trial for LQTS type 3, its further development has been ceased (20). The latest preclinical candidate for LQTS type 3 treatment is the small-molecule inhibitor of serum- and glucocorticoid-inducible kinase 1 (SGK1), which regulates cardiac *I*_Na_ and shortens APD in LQTS patient-derived iPSC-cardiomyocytes (21).

Hundreds of pathogenic variants in the Na_v_1.5-encoding *SCN5A* gene have been reported to underlie LQTS type 3. The first and most extensively characterized pathogenic variant is p.K1505_Q1507del or “ΔKPQ” (22, 23). Numerous patch-clamp studies in heterologous expression systems (CHO, HEK293, tsA201) transfected with mutant Na_v_1.5 channels have followed and contributed to our biophysical understanding of Na^+^ channels (17, 24, 25). Genetically engineered mice expressing LQTS type 3 mutant channels [the most common and prototypical variant DKPQ (or Scn5a^Δ/+^), but also hSC5A-N1325S] and the advent of iPS technology were instrumental in interpreting the genotype-phenotype correlation of the disease (21, 26–29). Collectively, all gain-of-function mechanisms increase the net inward Na^+^ current over the voltage range and time course of action potential, providing the substrate for arrhythmias (17, 23–25, 30). As a result, it is no surprise that drug development has primarily focused on the Na_v_1.5 channel as therapeutic target.

Here, we explored a different treatment approach prompted by previous encouraging results in LQTS type 2 and studied the therapeutic value of KCNQ1 antibodies in LQTS type 3. Out of six monoclonal antibody candidates, mAB-1 best performed as KCNQ1 channel agonist. With high binding affinity, it specifically targets the anticipated extracellular pore loop to open the KCNQ1 channel. The resulting K^+^ outflow (increased *I*_Ks_) shortened the cardiac repolarization phase in human iPSC-cardiomyocytes in a concentration-dependent manner. We then studied the effect of monoclonal KCNQ1 antibody in a cellular model of pharmacological LQTS type 3. Strikingly, mAB-1 normalized cardiac APD and showed antiarrhythmic properties. Mechanistically, monoclonal KCNQ1 antibody upregulates the repolarizing K^+^ *I*_Ks_ to counteract the pathologically increased late *I*_Na_ underlying LQTS type 3.

The present study is limited by the characterization of one monoclonal candidate, selected based on its electrophysiological properties best matching the previous results with polyclonal antibodies. At this point, we cannot exclude the potential therapeutic benefit of other monoclonal antibodies. From our prior work, we know that KCNQ1 antibodies are specific to *I*_Ks_ channels and do not have any impact on *I*_Na_ nor *I*_Kr_ (3). Nevertheless, the impact of mAB-1 on hERG and/or Na_v_1.5 channel expression remains unknown. It is also worth noting that in our study, we simulated LQTS type 3 using ATX-II in human iPSC-cardiomyocytes. As it is a pharmacological model, ATX-II can by no means fully replace gene-modulated or patient-derived iPSC-cardiomyocytes, which, in contrast, allows to interrogate distinct genetic backgrounds. Rather, the ATX-II model is considered a valuable surrogate for LQTS type 3, a readily available tool for drug screening and safety pharmacology in LQTS type 3. Although LQTS type 3 encompasses a varied spectrum of different Na^+^ channel gain-of-function mechanisms, it is intriguing that the ATX-II model reliably reproduces the main electrophysiological mutation traits, i.e., impaired fast inactivation leading to increased late *I*_Na_, augmented “window current,” slower current decay and faster recovery from inactivation (16, 17).

To conclude, we demonstrated that monoclonal KCNQ1 antibodies can correct an increased late *I*_Na_-mediated prolongation of cardiac repolarization through the increase of outward K^+^ current. Monoclonal KCNQ1 antibodies hold promise as the first-in-class antiarrhythmic treatment for LQTS. Nonetheless, the potential exists for on-target effects of KCNQ1 antibodies across multiple tissue types (31, 32). In the heart, a shortened atrial repolarization may predispose to atrial fibrillation, and we must be cognizant not to tilt the balance of ventricular repolarization toward a short QT syndrome. The broad extracardiac KCNQ1 channel expression may raise concerns of, e.g., hyperthyroidism (thyroid gland), diabetes mellitus (pancreas), and hypertension (adrenal cortex). As immunoglobulins are unlikely to cross the blood-brain or the blood-cochlear barrier, the risk for neuro-/ototoxicity should be low. Of note, in prior rabbit immunization studies no major adverse effects were observed (14). Future studies outlining the safety, pharmacokinetics, and pharmacodynamics of monoclonal KCNQ1 antibodies will then be decisive steps before entering a clinical trial.

## DATA AVAILABILITY

All data supporting this study are available upon request.

## SUPPLEMENTAL DATA

10.5281/zenodo.10049538 Supplemental Figs. S1–S3 and Supplemental Tables S1–S4: https://doi.org/10.5281/zenodo.10049538.

## GRANTS

This work was funded by Swiss National Science Foundation Grants Ambizione PZ00P3_173961 (to J.L.) and Eccellenza PCEFP3_203333 (to J.L.).

## DISCLOSURES

J.L. declares prior employment by BioMarin Pharmaceutical, Inc. The University of Bern holds a patent for the use of monoclonal antibody therapy in Long QT syndrome (WO 2023/031881), with J.L. listed as the inventor. The remaining author declares that the research was conducted in the absence of any commercial or financial relationships that could be construed as a potential conflict of interest.

## AUTHOR CONTRIBUTIONS

J.L. conceived and designed research; J.L. performed experiments; J.L. analyzed data; L.K. and J.L. interpreted results of experiments; J.L. prepared figures; L.K. and J.L. drafted manuscript; L.K. and J.L. edited and revised manuscript; L.K. and J.L. approved final version of manuscript.
